# Polypeptides Micelles Composed of Methoxy-Poly(Ethylene Glycol)-Poly(l-Glutamic Acid)-Poly(l-Phenylalanine) Triblock Polymer for Sustained Drug Delivery

**DOI:** 10.3390/pharmaceutics10040230

**Published:** 2018-11-13

**Authors:** Xingzheng Liu, Rongrong Fan, Boting Lu, Yuan Le

**Affiliations:** State Key Laboratory of Organic-Inorganic Composites, Beijing University of Chemical Technology, North Third Ring Road 15, Chaoyang District, Beijing 100029, China; 2016410002@mail.buct.edu.cn (X.L.); 2014210104@mail.buct.edu.cn (R.F.); 2016200044@mail.buct.edu.cn (B.L.)

**Keywords:** methoxy-poly(ethylene glycol), polypeptide, methoxy-poly(ethylene glycol)-poly(l-glutamic acid)-poly(l-phenylalanine), micelles, drug delivery

## Abstract

Methoxy-poly(ethylene glycol)-poly(l-glutamic acid)-poly(l-phenylalanine) triblock polymers with different architecture were synthesized as drug carrier to obtain sustained and controlled release by tuning the composition. These triblock polymers were prepared by ring opening polymerization and poly(ethylene glycol) was used as an initiator. Polymerization was confirmed by ^1^H NMR, FT-IR and gel penetration chromatography. The polymers can self-assemble to form micelles in aqueous medium and their critical micelle concentrations values were examined. The micelles were spherical shape with size of 50–100 nm and especially can arranged in a regular manner. Sorafenib was selected as the model drug and the drug loading performance was dependent on the composition of the block copolymer. In vitro drug release indicated that the polymers can realize controlled and sustained drug release. Furthermore, in vitro cytotoxicity assay showed that the polymers were biocompatible and the drug-loaded micelles can increase toxicity towards tumor cells. Confocal fluorescence microscopy assays illustrated that the micelles can be uptaken quickly and release drug persistently to inhibit tumor cell growth.

## 1. Introduction

According to the USA Food Drug Administration, the approved number of new drugs has declined to 25% in 2000–2010 compared with that in 1990–2000 [1]. Thus, maximizing the utilization of existing drug is an available method to manufacture high effective pharmaceutics. Developing new drug delivery system is a leading method to enhance the efficacy of active pharmaceutical ingredients, including poorly-water soluble compounds, ionic drugs, proteins, and peptides [2]. Polymer micelles is one of outstanding carriers used in anti-cancer drug delivery due to their excellent performance in enhancing drug solubility, prolonging drug circulation time and minimizing side effects [3]. Moreover, micelles can also release drug triggered by such as pH [3,4], temperature [5], ultrasound [6] and light [7].

Biodegradability and biocompatibility are the prime properties of polymer micelles as drug vehicles. Polypeptides, which are poly(amino acid)s linked by peptide bonds, are unique biodegradable and biocompatible polymers with structures mimicking natural proteins [8]. Amino acids can be not only ranged as different sequence or special structure [9], but also used as part of block and graft copolymers for preparing the core-shell structure micelles [10]. By appropriate modification of the molecule structure, a variety of polypeptide copolymers can be designed to possess varying degrees of hydrophobicity, structural attributes, and electrostatic properties [11]. Both poly(l-glutamic acid) (PLGA) and poly(l-phenylalanine) (PPA) are typical blocks utilized to form polypeptides as drug carriers [12]. Though PLGA-PPA copolymers have characteristic properties, their stability in the blood circulation is inadequate for the tumor targeting drug delivery. Due to the burst release or high drug release rate, the drug blood circulation time reduces. Poly(ethylene glycol) (PEG) is a non-toxic, non-immunogenic hydrophilic polymer with efficient function including preventing interactions with cells and proteins, inhibiting rapid clearance by the renal and reticuloendothelial systems and prolonging the circulation time in vivo [13]. It is used to stabilize complexes against self-aggregation in high concentrations and the absorption of proteins due to the cationic charges shield of PEG. In the case of micelles, the PEGylation of polypeptides positively influence its inherent shielding properties. Gref et al. firstly utilized PEG to protect the micelles from liver uptake and to increase blood circulation time in 1994 [14]. The introduction of the hydrophilic PEG is believed to reduce the toxicity and improve stability of the complex. 

Most of micelles are AB or ABA type copolymer, which lead to drug leakage due to their lack physical stability [15]. However, ABC triblock copolymer can effectively prevent these problems. Usually, for ABC triblock polymers, A block is hydrophilic segments, B block is backbone chains and C block is hydrophobic segments, separately. The ABC triblock copolymer can form core-shell-corona micelle structures, which sustain the polymer stable when environment changes [16]. Thus, the hydrophobic core serves as the reservoir for hydrophobic upload, and the hydrophilic corona stabilizes and protects the whole micelles [10]. Koo AN et al. prepared PEG-poly(l-lysine)-PPA triblock copolymer and studied drug loading and release profiles. Redox-sensitive poly(l-lysine) (PLys) layer can be applied to tumor targeting drug delivery system [17]. Zhou et al. synthesized polycaprolactone-PEG-*ε*-PLys triblock copolymer as multifunctional and biocompatible drug carrier with highly antimicrobial activity of Gram-negative and Gram-positive bacteria [18].

In this work, we designed a pH respondence mPEG-PLGA-PPA triblock copolymers to investigate the regularity between drug loading and release profiles and content of blocks. The polymer was synthesized via the ring opening polymerization (ROP) using PEG as an initiator. In the copolymer, PEG provides a compact steric protective corona layer to maintain the stability of micelles during biological circulation, and PLGA, as backbone chains, forms the shell of micelles. The hydrophobic PPA forms the core of micelles and was intended to allocate significant amounts of hydrophobic drugs and sustains the drug release. These copolymers can self-assemble to micelles in aqueous solution. Sorafenib (SFN), which is used for treating certain types of kidney and liver cancer, was chosen as the hydrophobic model drug. Influence of the content of PLGA and PPA block on drug loading and release performances were investigated. Furthermore, cell cytotoxicity and tumor cell uptake behavior of micelles have been explored to evaluate the potential for in vivo drug delivery. 

## 2. Materials and Methods 

### 2.1. Materials and Measurements

Methoxy-poly(ethylene glycol) (mPEG) (Mw = 2000), l-Glutamic acid and l-phenylalanine acid were purchased from Aladdin Industrial Corporation (Shanghai, China). SFN was purchased from Beijing Zhongshuo Pharmaceutical Technology Development Co., Ltd (Beijing, China). Benzyl-l-glutamate (BLG) was synthesized according to the literature procedure [19]. Anhydrous *N*,*N*-dimethylformamide (DMF), trifluoroacetic acid (TFA), HBr (33% in acetic acid), 3-(4,5-dimethyl-2-thiazolyl)-2,5-diphenyl-2-*H*-tetrazoliumbromide (MTT) and 4′,6-diamidino-2-phenylindole (DAPI) were purchased from Sigma-Aldrich Co. LLC (Shanghai, China). Tetrahydrofuran (THF) was supplied by Sinopharm Chemical Reagent (Beijing, China) and dried to remove water before use. All other solvents are of analytical grade and used without further purification.

^1^H NMR spectra was recorded on a Bruker Avance 400 spectrometer (Bruker Corporation, Karlsruhe, Germany) in dimethyl sulfoxide (DMSO)-*d*_6_ with 0.03% tetramethyl silane. Fourier transform-infrared spectroscopy (FT-IR) spectra was recorded on a Bruker VERTEX 70 instrument (Bruker Corporation) using the potassium bromide method. 

The critical micelle concentrations (CMC) of synthesized mPEG-PLGA-PPA was recorded on an Aminco Bowman Series 2 Luminescence Spectrometer (Spectrolab, Inc., Sylmar, CA, USA) by the fluorescence technique with a pyrene probe. In brief, a series of polymer solutions containing 6 × 10^−7^ M pyrene was prepared in distilled water. The solutions were equilibrated overnight at 20 °C and the emission wavelength was 373 nm for excitation spectra and excitation bandwidth was 5 nm. 

Dynamic laser scattering (DLS) measurements were performed on a Malvern ZS90 (Malvern Panalytical Ltd., Malvern, UK) at an angel of 173° and a temperature of 25 ± 0.1 °C. Scanning electron microscopy (SEM) measurements were performed on a JEOL JSM-7800F (JEOL co., Ltd., Tokyo, Japan) at an accelerating voltage of 5 kV. Molecular weight and molecular weight distributions were determined using an Agilent 2600 Series gel penetration chromatography (GPC) (Agilent Technologies, Inc., Santa Clara, CA, USA) instrument at 25 °C using DMF as the eluent. Ultraviolet-visible (UV-Vis) spectra was measured on a TU-1810 spectrophotometer (Puxi Corporation, Beijing, China). Circular dichroism spectra (CD) was performed from 190 nm to 300 nm at 25 °C. 

### 2.2. Synthesis of BLG-NCA, Phe-NCA, and Copolymer mPEG-PLGA-PPA

*γ*-benzyl-l-glutamate *N*-carboxyanhydride (BLG-NCA) and l-Phenylalanine *N*-carboxyanhydride (Phe-NCA) were prepared by triphosgene method [20]. BLG or l-Phenylalanine was dissolved in THF under nitrogen environment. Then, triphosgene was added into the mixture with continuously stirring at 50 °C. When the solution turned clear, the solvent was removed. The BLG-NCA (yield: 89.1%) or Phe-NCA (yield: 90.3%) was separated and purified by recrystallization with ethyl acetate and n-hexane.

**Figure 1:**
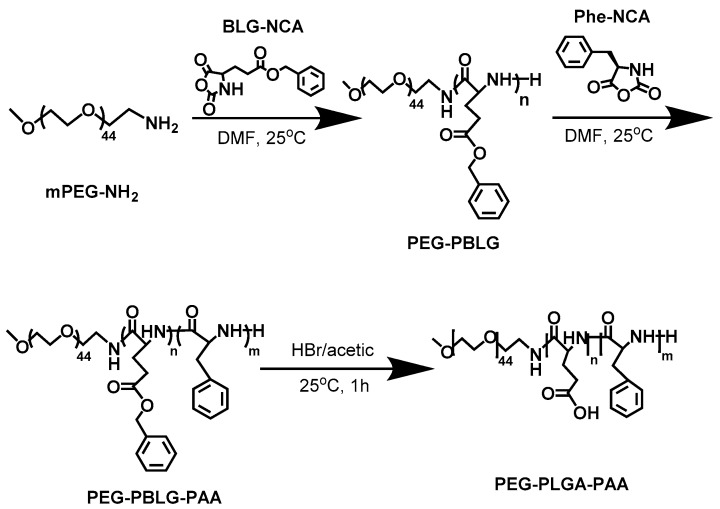
Synthetic scheme of mPEG-PLGA-PPA copolymer. PEG: poly(ethylene glycol); PLGA: poly(l-glutamic acid); PPA: poly(l-phenylalanine).

As shown in Figure 1, the triblock copolymer was synthesized in a two-stage process according to a procedure described in the literatures [21,22,23,24]. The proportional amount of BLG-NCA and Phe-NCA was dissolved in anhydrous DMF. Then, mPEG-NH_2_ (MW 2000) was added as the initiator of ROP and the polymerization was preceded for 72 h under N_2_ atmosphere to obtain complete reaction. After dialysis and lyophilizaton, mPEG-PBLG-PPA was obtained with a yield of 86.4%. Subsequently, mPEG-PBLG-PPA powder was dissolved in TFA. In order to hydrolyze the poly (*γ*-benzyl-l-glutamate) (PBLG), HBr was added with stirring for 1 h at room temperature. Finally, mPEG-PLGA-PPA powder was obtained by dialysis and lyophilizaton with a yield of 82.5%. 

### 2.3. Preparation of Blank and Drug-Loaded Micelles

The blank or SFN-loaded micelles were prepared according to a previous research [25]. In brief, 10 mg of mPEG-PLGA-PPA were dissolved in DMSO. After ultrasound irradiated, the solution was added dropwise to the deionized water under stirring. After 24 h, the solution was dialyzed (molecular weight cut-off 3500) and lyophilized to obtain blank micelles solution. To obtain SFN-loaded micelles, 2 mg of SFN was dissolved into DMSO with 10 mg mPEG-PLGA-PPA. Other steps were completely same. 

The lyophilized samples of SFN-loaded micelles were added into methanol and dissolved entirely by ultrasonic. The SFN concentration was determined by UV absorption intensity at 265 nm. The drug loading content (DLC) (wt%) and drug loading efficiency (DLE) (wt%) were calculated according to the following formulas.

(1) $$\left(\mathrm{DLC}\right)\left(\%\right)=\frac{\mathrm{amount}\;\mathrm{of}\;\mathrm{loaded}\;\mathrm{drug}}{\mathrm{amount}\;\mathrm{of}\;\mathrm{drug}-\mathrm{loaded}\;\mathrm{micelles}}\times 100\%\;$$

(2) $$\left(\mathrm{DLC}\right)\left(\%\right)=\frac{\mathrm{amount}\;\mathrm{of}\;\mathrm{loaded}\;\mathrm{drug}}{\mathrm{amount}\;\mathrm{of}\;\mathrm{feeding}\;\mathrm{drug}}\times 100\%\;$$

### 2.4. In Vitro Release Study

The weighed freeze-dried sample was put into a dialysis bag (MWCO = 3500 Da) and then dialyzed against the phosphate buffered saline solution (pH = 7.4) at 37 °C with continuous shaking at 100 rpm. At specific time intervals, 3 mL of the incubated solution was taken out and replaced with 3 mL fresh release medium. The concentration of the released SFN was determined by the UV absorption intensity at 265 nm. All measurements were conducted in triplicate.

### 2.5. In Vitro Cytotoxicity Assay

Cytotoxicity was measured by MTT assay [26]. HeLa cells were seeded in a 96-well plates at a density of 5000 cells per well in 100 μL of Dulbecco’s modified eagle medium (DMEM) containing 10% fetal bovine serum and cultured in an incubator (37 °C, 5% CO_2_) for 24 h. Then the cells were incubated with various concentrations of samples for 24 h. The cells were subjected to an MTT assay and were incubated for another 4 h. The supernatants were discarded, and 150 μL of DMSO was added to dissolve the generated formazan. The optical density (OD) was measured on a Multiskan MK3 microplate reader at 570 nm. The cell viability was calculated using the following equations and data were presented as means ± standard deviation (SD) (n = 3).

(3) $$\mathrm{Cell}\;\mathrm{viability}(\%)=\frac{{\mathrm{OD}}_{570,\mathrm{sample}}}{{\mathrm{OD}}_{570,\mathrm{control}}}\times 100\%\;$$

### 2.6. In Vitro Cellular Uptake

The cellular uptake was observed by confocal laser scanning microscopy (CLSM). The Hela cells were seeded on an 8-well FluoroDish^TM^ with 200 μL DMEM containing 10% fetal bovine serum and cultured in an incubator (37 °C, 5% CO_2_) for 12 h. Then the cells were incubated with coumarin-6 loaded micelles at a concentration of 2 μg/mL of coumarin-6. After 0.5 h, 4 h and 24 h incubation, the culture medium was removed and cells were washed three times with PBS. Then, the cells were fixed with 4% paraformaldehyde at room temperature, and the cell nuclei were stained with DAPI. The treated cells were visualized under a Leica TCS SP5 laser scanning confocal microscope. Excitation wavelengths of DAPI and coumarin-6 were 466 and 340 nm, respectively.

### 2.7. Statistical Analysis

All the values were presented as mean ± SD of at least three independent measurements. Statistical significance was tested by one-way ANOVA followed by a Student’s test for multiple comparison tests. Differences characterized by * *p* < 0.05 were considered statistically significant. 

## 3. Results and Discussion

### 3.1. Synthesis and Characterization of mPEG-PLGA-PPA

**Figure 2:**
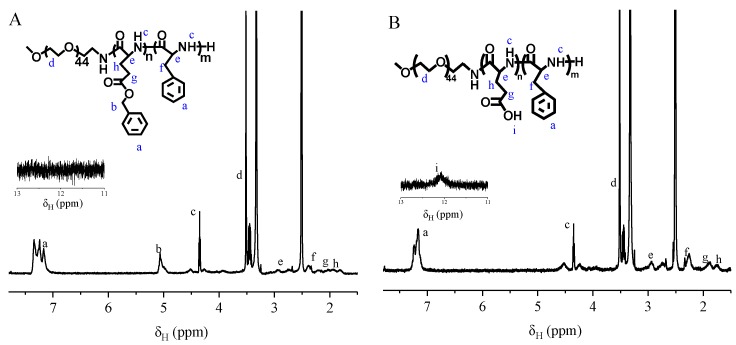
^1^H NMR of mPEG-poly(BLG)-PPA (**A**) and mPEG-PLGA-PPA (**B**) in DMSO-d_6._

mPEG-PLGA-PPA triblock copolymer was synthesized via the ROP of BLG-NCA and Phe-NCA. ^1^H nuclear magnetic resonance (NMR) spectra was shown in Figure 2. The multiplet at δ 7.167, 7.241 and 7.343 (a) were attributed to the protons on phenyl group. The singlet at δ 5.065 ppm (b) was assigned to the methylene attached to the phenyl group in PBLG block. The triplet (*J =* 5 Hz) at δ 4.351 ppm (c) corresponded to the protons in amido bond. The singlet at δ 3.514 ppm (d) was attributed to the methylene of PEG block. The multiplet at around δ 2.932 ppm (e) were assigned to the methine attached to the amido bond. The doublet (*J =* 6.4 Hz) at around δ 2.383 ppm (f) corresponded to the methylene attached to the phenyl group in PAA group. The triplet (*J =* 8.0 Hz) at δ 2.027 ppm (g) was attributed to the methylene attached to the ester group in PBLG block. The triplet (*J =* 7.0 Hz) at δ 1.064 ppm (h) was assigned to methylene attached to the main chain in PBLG group. The integral area ratio of a:b:c:d:e:f:g:h is 214:43:42:139:51:40:36:31. The deviation of integral area was mainly from the length distribution of block. The spectrum of PEG-PLGA-PAA was shown in Figure 2B. Most of peaks were similar except the PBLG block. Compared to the PEG-PBLG-PAA, the peaks at 7.343 corresponding to the phenyl group disappeared and the integral area at around 7.241 shrank to half its size. The peak assigned to methylene attached to the phenyl group in PBLG block also vanished. A singlet (i) emerged at δ 12.066 ppm, which was attributed to the protons in carboxylic acid group in PLGA block. The integral area ratio of i:a:c:d:e:f:g:h is 22:104:45:160:49:42:43:32. 

To calculate the molar ratio of each block in mPEG-PLGA-PAA, 3 peaks were chosen to represent every block. The peaks at around δ 3.517, 12.066, 7.250 ppm corresponded to PEG, PLGA and PPA block, respectively. The ratio was calculated with following equation:

$$n_{\mathrm{PEG}}:n_{\mathrm{PLGA}}:n_{\mathrm{PAA}}=\frac{A_{3.517}}{n_{3.517}}:\frac{A_{12.066}}{n_{12.066}}:\frac{A_{7.250}}{n_{7.250}}\;$$

Here, *n*_PEG_, *n*_PLGA_, *n*_PAA_ are the number of chain unit in PEG, PLGA, PAA block, respectively. *A*_3.517_ represents the integral area of peaks around 3.517 ppm, which have been chosen as the marker peaks of PEG block, and *n*_3.517_ represents the atom number in one PEG chain unit. The *A*_12.066_, *n*_12.066_, *A*_7.250_ and *n*_7.250_ have similar meaning for PLGA and PAA block.

**Figure 3:**
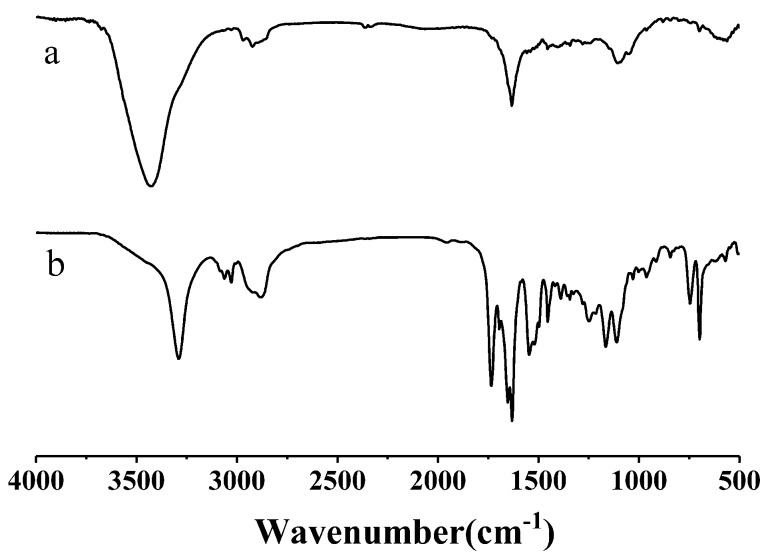
FT-IR spectra of copolymer mPEG-PLGA-PPA (a) and mPEG-poly(BLG)-PPA (b).

The FT-IR spectra of the copolymers are shown in Figure 3. The disappearance of the ester C=O stretching peak at 1725 cm^−1^ and the benzyl group signal at 7.36 ppm indicated the complete removing of the protecting group from the mPEG-PBLG-PPA. Both the ^1^H NMR and FT-IR spectra conclusively proved that the mPEG-PLGA-PPA triblock copolymer was successfully synthesized. The degree of polymerization was calculated from the peak integration ratio between the methylene protons of PEG, the phenyl proton signal of PPA and the methylene proton signal of PLGA.

The molecular weights of mPEG-PLGA-PPA were determined by GPC and listed in Table 1. It can also be seen that the number-average molar mass (Mn) of all the polymer products was in good agreement with the theoretical values. The polydispersity index (PDI) values were close to 1.0, indicating a well-controlled polymerization process and the effectiveness of purification, which is acceptable for further application of drug delivery.

### 3.2. Characterization of the Blank Micelles

The copolymers with hydrophilic PEG segment and hydrophobic PPA segment self-assembled to micelles in aqueous solutions. Using pyrene as a hydrophobic probe, the micelle formation of the copolymer can be proved by the measurement of CMC (Table 2). It can be seen that the CMC values of the copolymers were low, indicating that the copolymers could easily form into micelles in aqueous solution. Also, the CMC values decreased with increasing hydrophobic PPA content. The increase of PLGA hydrophilic block content could remarkably decrease CMC to 2.04 × 10^−3^ g/L. When the concentration of polymers rises, the repulsive force between the polymer and water will get larger than the attraction, resulting in the aggregation of the polymer propelled by van der Waals force. This repulsive force mainly derived from the hydrophobic groups, the attraction roots in the hydrophilic groups. This copolymer has two hydrophobic blocks, PEG and PLGA, which provide attraction to water, and one hydrophilic block, PAA, which afford repulsion to water. When the content of PAA block improves, the repulsion will be large and CMC values will decrease. 

**Figure 4:**
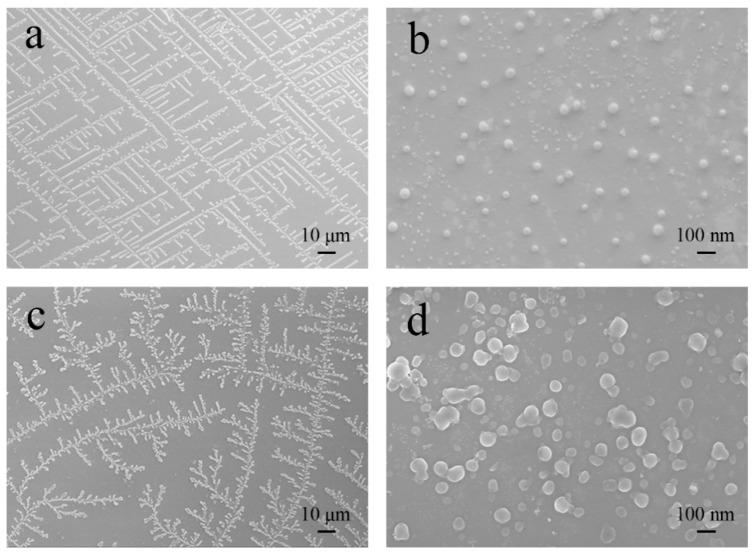
SEM micrographs of (**a**) mPEG_44_-PLGA_9_-PPA_14_ showing maze pattern, (**b**) mPEG_44_-PLGA_9_-PPA_14_, (**c**) mPEG_44_-PLGA_20_-PPA_18_ showing dendritic pattern and (**d**) mPEG_44_-PLGA_20_-PPA_18_.

The morphologies of the micelles were spherical shape with an average diameter of around 50~100 nm (Figure 4b,d). Especially, the micelles accordingly arranged in a regular way like “maze shape” (Figure 4a) or “dendritic shape” (Figure 4c) due to the self-assembly or crystallization of polypeptides molecules. Compared to mPEG_44_-PLGA_9_-PPA_14_, mPEG_44_-PLGA_20_-PPA_18_ used more reactant in synthesis, leading to a higher concentration and growth rate of micelles. Therefore, the size of mPEG_44_-PLGA_20_-PPA_18_ is larger than mPEG_44_-PLGA_9_-PPA_14_. Due to the larger size, mPEG_44_-PLGA_20_-PPA_18_ has a larger resistance in self-assemble and crystallization procedure. As a result, the mPEG_44_-PLGA_20_-PPA_18_ showed dendritic shape, which was a little less irregular than the maze shape.

**Figure 5:**
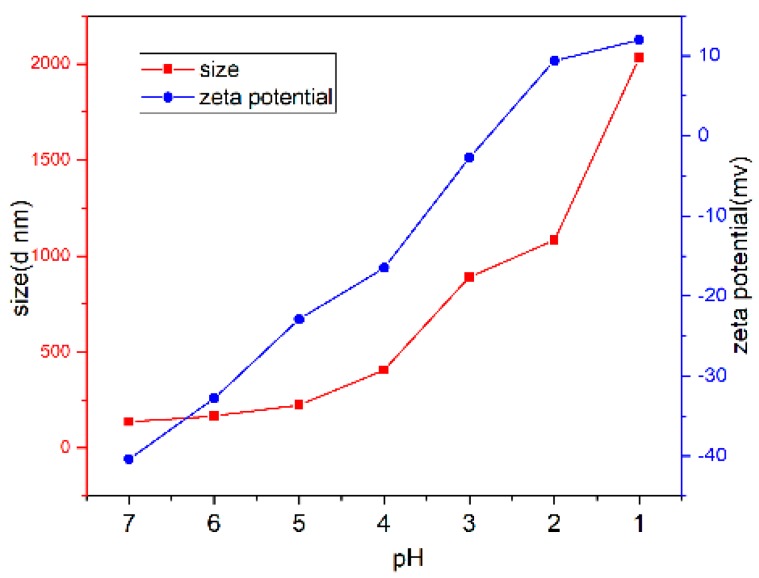
Size and zeta potential of polymeric micelles at different pH.

Due to demonstrate the pH response characteristics of mPEG-PLGA-PAA micelles, mPEG_44_-PLGA_9_-PPA_14_, which owned moderate content of PPA, was chosen to represent mPEG-PLGA-PAA micelles. The size and zeta potential of the polymeric micelles at different pH were measured by DSL and shown in Figure 5. The size of micelles significantly increased from 135 nm to 2031 nm as the pH decreased from 7 to 1, when pH < 5.0, the carboxyl groups in PLGA were in a fully deionized state, resulting in significant agglomeration and sharp increase of size. Similar pH-dependent agglomeration was also proved by the zeta potential, which dramatically increased from −40.4 mV to 12.5 mV at the pH range from 7 to 1.

**Figure 6:**
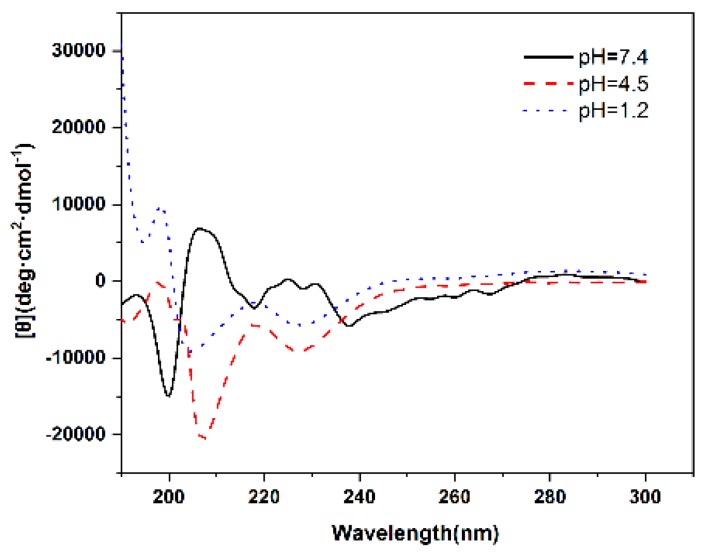
Circular dichroism spectra of mPEG-PLGA-PPA micelles at different pH.

The secondary structure of the polymer at different pH was performed using CD method (Figure 6). CD spectra can be approximated as a linear combination of secondary structure spectra. The secondary structure of polypeptide is mainly the interaction between local residues which is regulated by hydrogen bond. α helices, β sheets and random coil are the most important secondary structures in polypeptides [27]. The content of these secondary structures was estimated using the CONTINLL program and listed in Table 3. At pH 7.4, mPEG_44_-PLGA_9_-PPA_14_ mainly adopted a random coil (16.1%) and β sheets (82.5%) configuration, whereas exhibited α helices (43.8%) at pH 4.5 and the content of α helices increased to 66.9% at pH 1.2 due to protonation of molecules. Different content of secondary structure indicates the structural transformation of polymer. This transformation leads to the drug leakage, demonstrating that these micelles are pH-dependent.

### 3.3. Drug Loading Capacity and In Vitro Drug Release Behaviour 

Polymer architecture greatly influences drug encapsulation performance. DLC and DLE of micelles were measured and shown in Table 4. The drug was encapsulated in polymeric micelle core. Both PPA and SFN are water-insoluble, so the PPA content greatly influences the solubilization capacity of drug [28]. Therefore, the drug loading performance increased with increasing PPA content. However, much too high PPA content destroyed the balance of hydrophobic and hydrophilic portions, leading to instability and low solubilizing effect of SFN, so the DLC and DLE values of mPEG_44_-PLGA_9_-PPA_18_ micelles deceased to 6.3% and 43.0%. Due to more hydrophilic segment of PLGA, which balanced too much hydrophobic segments of the micelles, the DLC and DLE values of mPEG_44_-PLGA_20_-PPA_18_ micelles increased again. 

Table 5 illustrates the influence of drug and polymer weight ratios on DLC and DLE values of SFN-loaded micelles of mPEG_44_-PLGA_9_-PPA_14_ with the greatest drug carrying ability_._ It can be seen that the DLC and DLE values increased with the increase content of fed drug for drug-loaded micelles. The effect of the drug loading on size and zeta potential of micelles was studied by DLS. Zeta potential changed slightly. The average size was increased from 140.9 nm to 187.9 nm. The SFN-loaded particles are similar spheres as the particles without SFN, while the size increases a little. However, when the ratio reached to 1:5, the DLC and DLE values decreased slightly, showing the drug loading was approaching saturation. 

When drugs are physically encapsulated in stable polymeric micelles, the drug release rate is controlled by the diffusion out of the micelle core and/or by dissociation of the micelles. Lavasanifar et al. addressed some factors to control the drug release from micelles [29]. The diffusion rate may be quite low if the drug prefers to interact with the core-forming block. The release rate of the drug from the micelles is accelerated with an increased content of PEG but delayed with more hydrophobic chains [30]. The localization of the solute in the core/shell structure, micellar size and molecular volume of the drug are other factors that also influence the rate of drug diffusion from the polymeric carrier. 

**Figure 7:**
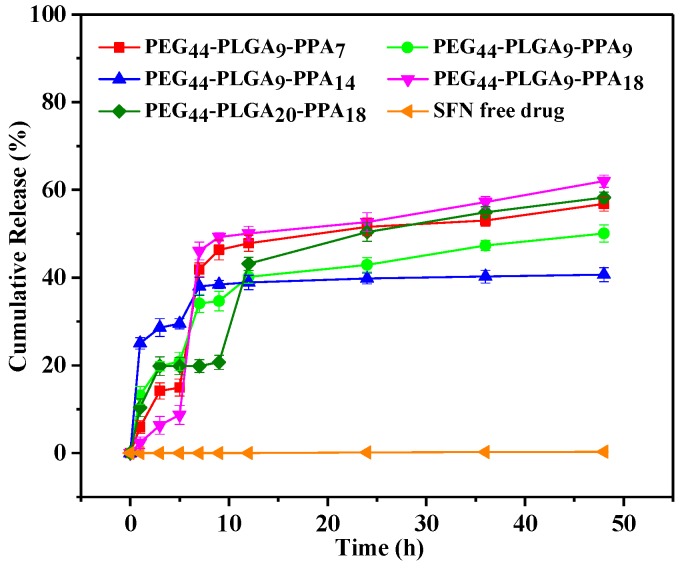
In vitro drug release profiles of SFN loaded mPEG-PLGA-PPA micelles at pH 7.4.

The in vitro release behaviors of SFN from mPEG-PLGA-PPA micelles were evaluated using a dialysis method at 37 °C in release media of pH 7.4. This medium with very low saturated solubility of SFN was chosen to measure the enhancing ability of SFN dissolution. As shown in Figure 7, SFN loaded micelles shows faster and more drug release than free SFN. The release details were shown in Table 6. The drug release profiles showed quick release after 1 h and then slowly rising. After 1 h, the release amount was no more than 30%, indicating there is no burst release. At this point, the majority of drug loaded by physical absorption had released from the micelles. Except mPEG_44_-PLGA_20_-PPA_18_, polymers with balanced ratio of PLGA and PPA release more drug in burst release. This balanced ratio is between 1 and 1.5. When the polymer chain was lengthened, the burst release reduced. In 1–12 h, drug release shows a slow release period. Polymers with different block ratio show different platforms. Owing to the ABC triblock structure, SFN is embedded in the hydrophobic core. This part of drug won’t release until the polymer eroded. Longer chain is hard to disperse in water, so mPEG_44_-PLGA_20_-PPA_18_ has longer platform than others. Then, along with the PEG layer disperses, the PLGA layer exposes, resulting a rapid release after platform. After 12 h, release was very slowly because the remaining SFN was protected by PLGA and PPA blocks. Polymers with balanced ratio store more drug in hydrophobic core, so at the end of drug release test, these polymers release less drug especially mPEG_44_-PLGA_9_-PPA_14_. 

### 3.4. In Vitro Cytotoxicity and Cellular Uptake Behavior

**Figure 8:**
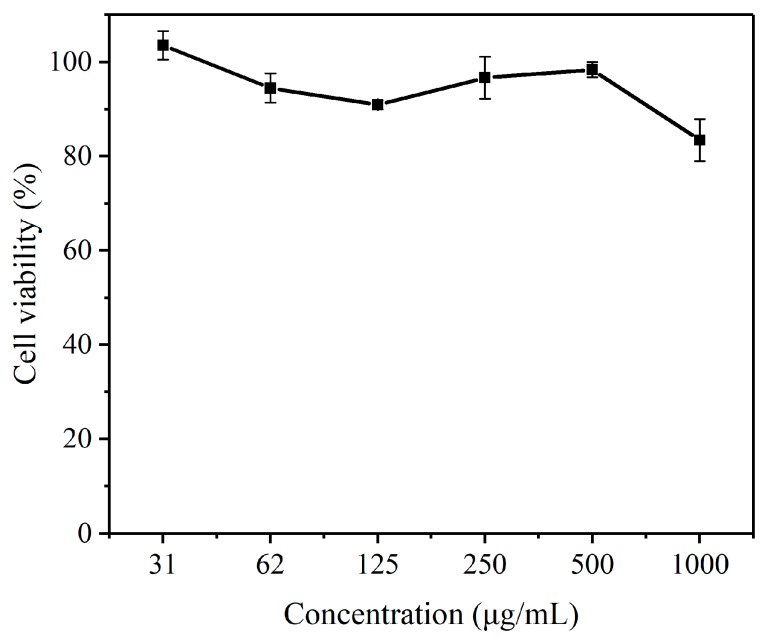
Cell viabilities of HeLa cells incubated with polymer micelles for 24 h (n = 3, mean ± SD).

Figure 8 showed the micelles of copolymer mPEG-PLGA-PPA had low cytotoxicity. The cell viability (%) of HeLa cells in the presence of various concentrations of blank micelles was over 80% for mPEG_44_-PLGA_9_-PPA_14_ (31–1000 μg/mL). The results revealed acceptable biocompatibility and low cytotoxicity of mPEG-PLGA-PPA micelles.

**Figure 9:**
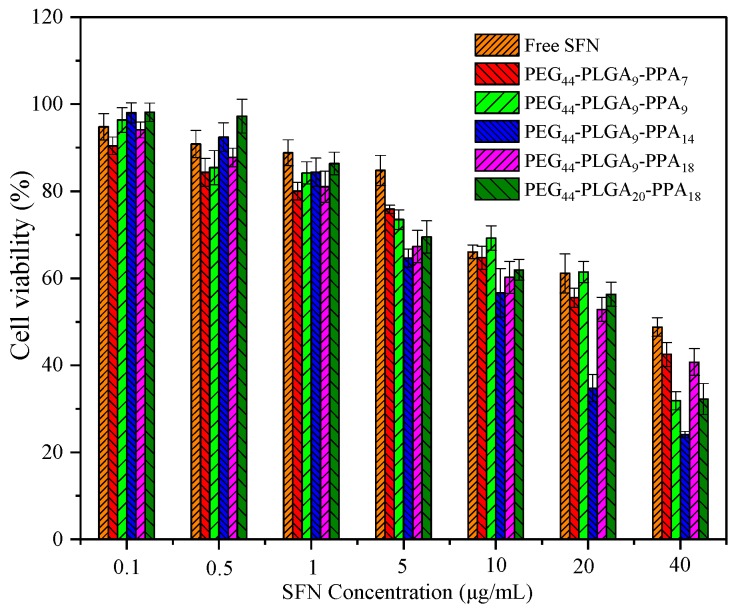
Cell viabilities of HeLa cells incubated with free SFN and SFN-loaded micelles for 24 h (n = 3, mean ± SD).

Figure 9 showed the inhibition effects of SFN free drug and drug loaded micelles against HeLa cells. After 24 h, both free drug and drug-loaded micelles showed dose-dependent cell inhibition behavior. For blank micelles, their cell viability slightly decreased with increasing of hydrophobic contents. For SFN loaded micelles, their IC_50_ values vary with their DLC, and are always lower than that of free SFN. The increasing inhibition effect of SFN free drug against HeLa cells may due to more uptake by HeLa cells.

**Figure 10:**
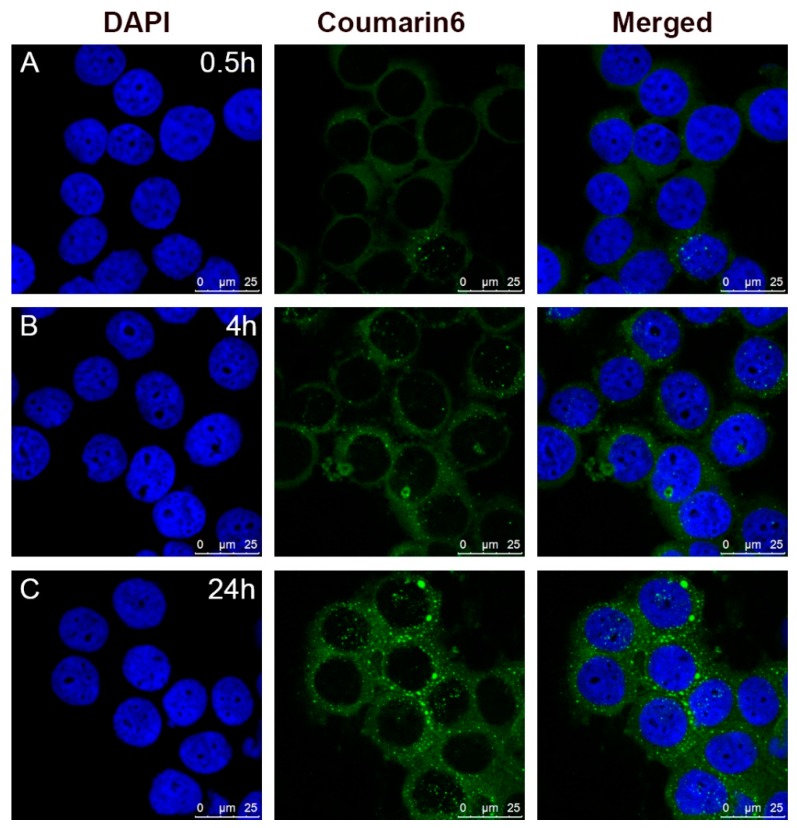
Confocal laser scanning microscopy (CLSM) images of HeLa cells incubated with coumarin-6 labeled drug loaded micelles after (**A**) 0.5 h, (**B**) 4 h and (**C**) 24 h.

The cellular uptake and intracellular release behavior of coumarin-6 loaded micelles in HeLa cells were investigated by CLSM. As shown in Figure 10, coumarin-6 emerged in the cell cytoplasm after 0.5 h. After 4 h, more green fluorescence of coumarin-6 could be observed in the cell cytoplasm and distributed widely in the cytoplasm, indicating that the micelles with coumarin-6 could be taken into cells quickly and effectively. After 24 h, the fluorescence in cytoplasm become stronger, almost up to the most level, showing a sustained intracellular release of loaded drug.

## 4. Conclusions

Triblock copolymers of mPEG-PLGA-PPA with different architecture were successfully synthesized via ROP. The self-assembled polymeric micelles exhibited pH sensitivities. SFN was loaded into the copolymers and the DLC in vitro release behavior of SFN from micelles can be tuned by adjusting the hydrophilic/hydrophobic ratios of the polymer. The cytotoxicity studies showed that the polymers were non-toxic and drug-loaded micelles exhibited an increasing antitumor effect compared with SFN free drug. CLSM images indicated a fast internalization of the drug loaded micelles and long-lasting intracellular drug release. Consequently, we propose the mPEG-PLGA-PPA copolymers as potential controlled intracellular drug delivery carriers.

## Figures and Tables

**Table 1:** Molecular weight of mPEG-PLGA-PPA copolymers.

Polymers	Feed Molar Ratio ^a^	Resultant Molar Ratio ^b^	Mn, GPC ^c^ (g/mol)	Mn, Th ^d^ (g/mol)	PDI, GPC ^c^
mPEG_44_-PLGA_9_-PPA_7_	1/0.8	1/0.9	5398	4237	1.06
mPEG_44_-PLGA_9_-PPA_9_	1/1.0	1/1.2	5550	4502	1.04
mPEG_44_-PLGA_9_-PPA_14_	1/1.5	1/1.6	6341	5237	1.04
mPEG_44_-PLGA_9_-PPA_18_	1/2.0	1/2.1	7432	5825	1.08
mPEG_44_-PLGA_20_-PPA_18_	1/0.9	1/1.1	8095	7266	1.12

^a^ Feed molar ratio of BLG-NCA/Phe-NCA. ^b^ Resultant molar ratio of PLGA/PPA calculated by ^1^H NMR. ^c^ Determined by gel penetration chromatography (GPC). ^d^ Theory values.

**Table 2:** Critical micelle concentration (CMC) values of mPEG-PLGA-PPA copolymers.

Polymers	CMC (g/L)
mPEG_44_-PLGA_9_-PPA_7_	2.12 × 10^−2^
mPEG_44_-PLGA_9_-PPA_9_	1.98 × 10^−2^
mPEG_44_-PLGA_9_-PPA_14_	1.84 × 10^−2^
mPEG_44_-PLGA_9_-PPA_18_	1.53 × 10^−2^
mPEG_44_-PLGA_20_-PPA_18_	2.04 × 10^−3^

**Table 3:** Secondary conformation of polymer micelles at different pH.

Polymers	pH	Helices (%)	Sheets (%)	Random Coil (%)
mPEG_44_-PLGA_9_-PPA_14_	7.4	0.0	82.5	16.1
	4.5	43.8	11.8	26.8
	1.2	66.9	20.1	0.0

**Table 4:** Drug loading profiles of mPEG-PLGA-PPA copolymers with different block ratios.

Polymers	Feed Molar Ratio ^a^	Resultant Molar Ratio ^b^	DLC (%)	DLE (%)
mPEG_44_-PLGA_9_-PPA_7_	1/0.8	1/0.9	6.7	44.2
mPEG_44_-PLGA_9_-PPA_9_	1/1.0	1/1.2	9.1	54.4
mPEG_44_-PLGA_9_-PPA_14_	1/1.5	1/1.6	11.2	72.1
mPEG_44_-PLGA_9_-PPA_18_	1/2.0	1/2.1	6.3	43.0
mPEG_44_-PLGA_20_-PPA_18_	1/0.9	1/1.1	8.9	52.1

^a^ Feed molar ratio of BLG-NCA/Phe-NCA. ^b^ Resultant molar ratio of PLGA/PPA calculated by ^1^H NMR.

**Table 5:** Drug loading content (DLC) and drug loading efficiency (DLE) of Sorafenib (SFN)-loaded assembled micelles of different drug/carrier mass ratio.

Polymer	Drug/Carrier (Mass Ratio)	DLC (%)	DLE (%)	Size (nm)	Zeta Potential (mv)
mPEG_44_-PLGA_9_-PPA_14_	1:5	11.5	70.3	179.8	−29.5
	1:6	13.0	75.6	187.9	−29.9
	1:7	12.1	73.3	164.2	−33.0
	1:8	11.2	72.1	140.9	−34.2

**Table 6:** In vitro drug release profiles of SFN loaded mPEG-PLGA-PAA micelles.

Polymer	Burst Release	Release at 12 h	Release at the End	Plateau Time	Plateau Values
mPEG_44_-PLGA_9_-PPA_7_	6.0%	47.8%	56.8%	9–12 h	47.1%
mPEG_44_-PLGA_9_-PPA_9_	13.2%	40.2%	50.1%	7–9 h	34.4%
mPEG_44_-PLGA_9_-PPA_14_	25.0%	38.9%	40.7%	none	-
mPEG_44_-PLGA_9_-PPA_18_	2.4%	50.1%	62.%	none	-
mPEG_44_-PLGA_20_-PPA_18_	10.3%	43.2%	58.3%	3–9 h	20.1%

## References

[1] Cohen F.J. (2005). Macro trends in pharmaceutical innovation. Nat. Rev. Drug Discov..

[2] Mahapatro A., Singh D.K. (2011). Biodegradable nanoparticles are excellent vehicle for site directed in-vivo delivery of drugs and vaccines. J. Nanobiotechnol..

[3] Li B.Q., Shan M., Di X., Gong C., Zhang L.H., Wang Y.M., Wu G.L. (2017). A dual pH- and reduction-responsive anticancer drug delivery system based on PEG-SS-poly(amino acid) block copolymer. RSC Adv..

[4] Folchman-Wagner Z., Zaro J., Shen W.C. (2017). Characterization of polyelectrolyte complex formation between anionic and cationic poly(amino acids) and their potential applications in pH-dependent drug delivery. Molecules.

[5] Lu D.D., Zhang Y.Y., Li T., Li Y.F., Wang H.S., Shen Z.Q., Wei Q.B., Lei Z.Q. (2016). The synthesis and tissue adhesiveness of temperature-sensitive hyperbranched poly(amino acid)s with functional side groups. Polym. Chem.

[6] Cochran M.C., Eisenbrey J.R., Soulen M.C., Schultz S.M., Ouma R.O., White S.B., Furth E.E., Wheatley M.A. (2011). Disposition of ultrasound sensitive polymeric drug carrier in a rat hepatocellular carcinoma model. Acad. Radiol..

[7] Gong C., Lu C.C., Li B.Q., Shan M., Wu G.L. (2017). Dopamine-modified poly(amino acid): An efficient near-infrared photothermal therapeutic agent for cancer therapy. J. Mater. Sci..

[8] Deming T.J. (2016). Synthesis of side-chain modified polypeptides. Chem. Rev..

[9] Cerrato C.P., Lehto T., Langel U. (2014). Peptide-based vectors: Recent developments. Biomol. Concepts.

[10] Xu H., Yao Q., Cai C., Gou J., Zhang Y., Zhong H., Tang X. (2015). Amphiphilic poly(amino acid) based micelles applied to drug delivery: The in vitro and in vivo challenges and the corresponding potential strategies. J. Control. Release.

[11] Lin J., Zhang S., Chen T., Lin S., Jin H. (2007). Micelle formation and drug release behavior of polypeptide graft copolymer and its mixture with polypeptide block copolymer. Int. J. Pharm..

[12] Shen Y., Fu X., Fu W., Li Z. (2015). Biodegradable stimuli-responsive polypeptide materials prepared by ring opening polymerization. Chem. Soc. Rev..

[13] Du Z., Pan S.R., Yu Q., Li Y.P., Wen Y.T., Zhang W., Feng M., Wu C.B. (2010). Paclitaxel-loaded micelles composed of folate-poly(ethylene glycol) and poly(gamma-benzyl l-glutamate) diblock copolymer. Colloid Surf. A.

[14] Gref R., Minamitake Y., Peracchia M.T., Trubetskoy V., Torchilin V., Langer R. (1994). Biodegradable long-circulating polymeric nanospheres. Science.

[15] Ko J., Park K., Kim Y.S., Kim M.S., Han J.K., Kim K., Park R.W., Kim I.S., Song H.K., Lee D.S. (2007). Tumoral acidic extracellular pH targeting of pH-responsive MPEG-poly(beta-amino ester) block copolymer micelles for cancer therapy. J. Control. Release.

[16] Hadjichristidis N., Iatrou H., Pitsikalis M., Pispas S., Avgeropoulos A. (2005). Linear and non-linear triblock terpolymers. Synthesis, self-assembly in selective solvents and in bulk. Prog. Polym. Sci..

[17] Koo A.N., Lee H.J., Kim S.E., Chang J.H., Park C., Kim C., Park J.H., Lee S.C. (2008). Disulfide-cross-linked PEG-poly(amino acid)s copolymer micelles for glutathione-mediated intracellular drug delivery. Chem. Commun..

[18] Zhou C.C., Zhou X.Y., Su X.K. (2017). Noncytotoxic polycaprolactone-polyethyleneglycol- epsilon-poly(l-lysine) triblock copolymer synthesized and self-assembled as an antibacterial drug carrier. RSC Adv..

[19] Blout E.R., Karlson R.H. (1956). Polypeptides. III. The synthesis of high molecular weight poly-γ-benzyl-l-glutamates1. J. Am. Chem. Soc..

[20] Wang W.L., Zhang L., Liu M.T., Le Y., Lv S.S., Wang J.X., Chen J.F. (2016). Dual-responsive star-shaped polypeptides for drug delivery. RSC Adv..

[21] Neal J.C., Stolnik S., Schacht E., Kenawy E.R., Garnett M.C., Davis S.S., Illum L. (1998). In vitro displacement by rat serum of adsorbed radiolabeled poloxamer and poloxamine copolymers from model and biodegradable nanospheres. J. Pharm. Sci..

[22] Sanson C., Schatz C., Le Meins J.F., Brulet A., Soum A., Lecommandoux S. (2010). Biocompatible and biodegradable poly(trimethylene carbonate)-b-poly(l-glutamic acid) polymersomes: Size control and stability. Langmuir.

[23] Kim J.O., Oberoi H.S., Desale S., Kabanov A.V., Bronich T.K. (2013). Polypeptide nanogels with hydrophobic moieties in the cross-linked ionic cores: Synthesis, characterization and implications for anticancer drug delivery. J. Drug Target..

[24] Li J., Li J., Xu S., Zhang D., Liu D. (2013). Hydrophobic oligopeptide-based star-block copolymers as unimolecular nanocarriers for poorly water-soluble drugs. Colloids Surf. B Biointerfaces.

[25] Jeong Y.I., Seo S.J., Park I.K., Lee H.C., Kang I.C., Akaike T., Cho C.S. (2005). Cellular recognition of paclitaxel-loaded polymeric nanoparticles composed of poly(gamma-benzyl l-glutamate) and poly(ethylene glycol) diblock copolymer endcapped with galactose moiety. Int. J. Pharm..

[26] Hansen M.B., Nielsen S.E., Berg K. (1989). Re-examination and further development of a precise and rapid dye method for measuring cell growth/cell kill. J. Immunol. Methods.

[27] Zhang L., Zhang P., Zhao Q., Zhang Y., Cao L., Luan Y. (2016). Doxorubicin-loaded polypeptide nanorods based on electrostatic interactions for cancer therapy. J. Colloid Interface Sci..

[28] Qiu L.Y., Bae Y.H. (2006). Polymer architecture and drug delivery. Pharm. Res..

[29] Lavasanifar A., Samuel J., Kwon G.S. (2002). Poly(ethylene oxide)-block-poly(l-amino acid) micelles for drug delivery. Adv. Drug Deliv. Rev..

[30] Ahmed F., Discher D.E. (2004). Self-porating polymersomes of PEG-PLA and PEG-PCL: Hydrolysis-triggered controlled release vesicles. J. Control. Release.

